# Forest 404: Using a BBC drama series to explore the impact of nature’s changing soundscapes on human wellbeing and behavior

**DOI:** 10.1016/j.gloenvcha.2022.102497

**Published:** 2022-05

**Authors:** Alexander J. Smalley, Mathew P. White, Rebecca Ripley, Timothy X Atack, Eliza Lomas, Mike Sharples, Peter A. Coates, Nick Groom, Ann Grand, Ailish Heneberry, Lora E. Fleming, Michael H. Depledge

**Affiliations:** a University of Exeter, UK; b University of Vienna, Austria; c British Broadcasting Corporation, UK; d Sleepdogs.org, UK; e The Open University, UK; f University of Bristol, UK; g University of Macau, China; h Eden Project International, UK

**Keywords:** Environmental psychology, Attention restoration, Wellbeing, Soundscape, Conservation behavior, Ecosystem services, Biodiversity loss

## Abstract

Extensive ecosystem degradation and increasing urbanization are altering human relationships with nature. To explore these trends, we created a transdisciplinary, narrative-led podcast series produced by the BBC, called *Forest 404*. The series explored the implications of a world without nature. An online experimental component mobilized audience participation (n = 7,596) to assess responses to natural soundscapes with and without abiotic, biotic, and poetic elements across five biomes. Conditions featuring the sounds of wildlife, such as bird song, were perceived to be more psychologically restorative than those without. Participants’ personal lived experiences were strongly related to these outcomes; those who had memories triggered by the sounds were more likely to find them psychologically restorative and exhibited a greater motivation to preserve them. Moreover, the effects of both soundscape composition and memories on preservation behavior were partially mediated by restorative potential; respondents were more likely to want to protect the sounds they heard if they thought they might offer therapeutic outcomes. Our findings highlight the value of art-science collaborations and demonstrate how maintaining contact with the natural world can promote wellbeing and foster behaviors that protect planetary health.

## Highlights

• A novel and transdisciplinary eco-thriller podcast series was produced by the BBC.
• An integrated experiment explored appraisals of changing natural soundscapes.
• Participants responded differently to soundscapes with and without audible wildlife.
• Lived experience moderated both restorative potential and preservation motivation.
• Perceived therapeutic qualities partly mediated desires to preserve natural sounds.

## 1 Introduction

The planet is undergoing wholesale ecological degradation, with estimates of accelerating environmental decline abound: climate change is increasing the destruction of natural habitats (Travis, 2003); anthropogenic materials now contaminate the land, freshwaters, seas, and air (Rochman and Hoellein, 2020); and global reductions in biodiversity (Newbold et al., 2016) are unfolding at rates fast enough to herald a sixth mass extinction event (Ceballos et al., 2015). These trends are exceeding earth’s planetary boundaries (Rockstrom et al., 2009), catalyzing the development of global pandemics (IPBES, 2020), and causing the widespread collapse of natural systems (Bergstrom et al., 2021).

This trajectory clearly matters if human populations are to not just survive but thrive. Robust and functioning ecosystems provide many services vital for human health, such as clean air, fresh water, and climate regulation (Millennium Ecosystem Assessment, 2005). A significant body of evidence also suggests that safe, constructive contact with the natural world is important for a wide range of positive physical and mental health outcomes (Frumkin et al., 2017). In particular, exposure to nature can reduce stress (Ward Thompson et al., 2012), help people cope with challenging situations (Lederbogen et al., 2011), support cognitive functioning and emotional wellbeing (Bratman et al., 2019), and reduce negative rumination, a key risk factor in depression (Bratman et al., 2015).

However, despite public messaging designed to raise awareness of the consequences of an ailing natural environment (WWF, 2020), collective action to redress global trends has been slow to materialize. This inertia may in part stem from the fact that increasingly few people are present to witness environmental crises firsthand; over 55% of the world’s human inhabitants now reside in urban environments – a figure projected to reach 68% by 2050 and which is already above 81% in higher-income regions such as North America (United Nations, 2019). These demographic shifts are reducing opportunities for direct contact with natural settings and biodiverse settings in particular (Turner et al., 2004), leading to worries about how an ‘extinction of experience’ might affect public health and influence societal attitudes towards environmental protection (Soga and Gaston, 2016). Moreover, visualizing the consequences of ecological change can be challenging for people (Pahl and Bauer, 2011), limiting the effectiveness of scientific approaches designed to communicate potential environmental futures (Sheppard, 2012).

To address these shortcomings, there have been increasing calls for the formation of academic and creative alliances that engage wide audiences with scientific findings (Hess et al., 2020) and reconnect urban communities to the natural world (Ives et al., 2018). Fictional literature has emerged as an encouraging tool in this endeavor, employing creative storytelling as a successful way to involve the public in modern ecological issues (Schneider-Mayerson et al., 2020). Here we extend upon these methods, embracing the scientific communication potential of Web 2.0 (Brossard and Scheufele, 2013) via the medium of audio podcasting.

Through a multi-institution arts and science collaboration we developed a podcast series, titled *Forest 404*, that engaged audiences with environmental issues and mobilized their participation in a large online experiment (n = 7,596). The premise of the series suggested that humans have an intrinsic and hard-wired affective response to the sounds of nature (Wilson, 1984). Our experimental approach probed the assumption that all participants would respond the same to differing environmental sounds, and we present findings that challenge this hypothesis. We first introduce the *Forest 404* series, outline the key elements of our study design, then present our results and conclusions.

### 1.1 The Forest 404 series

Named after the error message encountered when searching for a web page that no longer exists, *Forest 404* was written by Timothy X Atack and produced by the UK’s national broadcaster, the British Broadcasting Corporation (BBC). The series was based around a nine-part ‘eco-thriller’, set in a not-too-distant future, where ecological trends have resulted in the eradication of natural environments, the technological replacement of ecosystem services, and an elimination of nature from cultural history.

Episode one introduced Pan, the series’ protagonist played by Pearl Mackie, at her job in a data archiving facility. Data storage was at a premium in this future society and Pan was charged with prioritizing ‘ancient’ audio files for preservation or deletion; a task she had little trouble negotiating until she was presented with the soundscape of a tropical rainforest. Pan was beguiled by the audio but unable to relate it to the world in which she lived. *Forest 404* followed Pan’s reaction to the sounds of nature and explored how losing a connection to natural environments could impact both planetary and human health (visit http://bbc.co.uk/forest to listen in full).

### 1.2 Series engagement

The *Forest 404* series sought to harness the capacity of landmark BBC nature programs to engage large audiences in environmental issues (BBC News, 2018). Each episode of the drama was accompanied by an interview-based talk that explored issues covered in the fiction with topic experts; an immersive natural soundscape for listeners to engage with; and a statement encouraging participation in the online experiment. This novel cross-genre format created 27 podcast episodes that were released in April 2019 through internet browsers, the BBC Sounds smartphone app, and international podcast services (such as iTunes and Spotify). Between April 2019 and March 2020, episode downloads exceeded 2.5 million. The series won accolades from Prix Europa, the UK Writers’ Guild, and the Audio and Radio Industry Awards.

### 1.3 Research focus

In synergy with the narrative and audio-based format of *Forest 404*, our experiment used sound to probe participant responses to natural environments. Our primary focus was on how varying natural soundscapes might provide ‘perceived restorative potential’, an indirect measure of the bottom-up recovery of positive attentional and affective states posited by Attention Restoration Theory (Kaplan, 1995). This multifaceted construct captures assessments of how restorative an environment is believed to be (Hartig et al., 1997, Payne, 2013) and is commonly used alongside scenarios that ask participants to imagine a time of diminished cognitive resources, such as after a long day at work, or following time spent in a busy, noisy, urban environment (Ratcliffe et al., 2016, Staats and Hartig, 2004). Our second focus was a simulation of participant behavior. We asked respondents to imagine they were Pan, *Forest 404′s* lead character, and make decisions to ‘keep’ the sounds they were presented with in an archive, or ‘delete’ them to make space for other data. The question was a direct analogue for the choices Pan faces in the drama, worded so those who had not listened to the series could also respond effectively. We refer to this behavior here as ‘preservation motivation’ (Prince, 1989), and concentrate on incentives to conserve natural capital that reflect nature-first priorities (e.g. protection of rare species), or human-first priorities (e.g. wellbeing benefits); factors that strongly overlap with the motives underpinning pro-environmental behaviors (Dearborn and Kark, 2010, Gifford and Nilsson, 2014) and align with the broad notion of soundscape conservation (Dumyahn and Pijanowski, 2011).

### 1.4 The importance of sound

Our emphasis on soundscapes addressed a gap in the existing literature, which has overwhelmingly centered on the visual perception of natural settings. For example, studies of psychological restoration have often relied upon still and moving images as their exposure conditions (Korpela, 2013); focused on aesthetic properties such as view and composition (Gatersleben and Andrews, 2013, Kaplan, 2001); or assessed contrasts between urban and natural scenes (Van den Berg et al., 2016). However, sound represents an intrinsic mechanism through which nature is sensed and experienced (Conniff and Craig, 2016, Fisher, 1999).

The impacts of anthropogenic sound have been extensively studied under the rubric of noise pollution (Murphy and King, 2014) with the presence of audible factors such as mechanized industry and transport demonstrating detrimental effects on landscape experience (Miller, 2008). Nevertheless, mitigating unwanted acoustic elements to achieve a state of ‘quiet’ may not automatically lead to the positive appraisal of a soundscape (Brown, 2015). Attention has instead turned to how nature-based sounds might contribute to the idea of ‘natural quiet’ (Brown, 2012), a shift in focus that values natural soundscape components (rather than the lack thereof) as positive resources (Kang et al., 2016). In this vein, research across several disciplines has identified a consistent set of preferences for acoustic sources (see Ratcliffe (2021) for a comprehensive review). For example, listening to the soundscape of the natural world is almost always preferred to that of urban environments (Alvarsson et al., 2010, Benfield et al., 2014, Schafer, 1977, Uebel et al., 2021), with elements such as flowing water (Carles et al., 1999, Yang and Kang, 2005) and passerine birdsong (Hedblom et al., 2014, Ratcliffe et al., 2016) commonly receiving high appraisal ratings.

Soundscapes featuring these components have also demonstrated the potential to reduce the physiological and psychological indices of stress, facilitate recovery from cognitive fatigue, and increase positive emotional states (Buxton et al., 2021, Ratcliffe, 2021). This therapeutic potential has been attributed to several theoretical mechanisms that may operate concurrently, most notably: adaptive, evolutionary processes where natural quiet might signify a place suitable to ‘rest and digest’ (Andringa and Bosch, 2013, Gould van Praag et al., 2017); an extension of Attention Restoration Theory (Kaplan, 1995), in which natural sounds stimulate feelings of fascination and ‘being away’ that might facilitate the recovery of attentional resources (Payne, 2013); and also top-down mechanisms through which acoustic stimuli might trigger memories and associations capable of encouraging psychological restoration (Gould van Praag et al., 2017, Haga et al., 2016, Ratcliffe et al., 2016).

Sound is thus emerging as an essential ingredient in restorative nature-based experiences (Annerstedt et al., 2013). Yet sound sources such as a singing blackbird or babbling brook clearly differ in origin, distribution, temporality, and meaning. Could these kinds of contrasting sound types confer differential restorative advantages, and how might their combinations produce additive or competing effects?

The dissection of soundscapes according to their constituent components has been expanded by the field of acoustic ecology, which commonly distinguishes between geophysical, biological, and anthropogenic sources (Pijanowski et al., 2011). These approaches have presented novel ways to assess fluxes in audible fauna through sonic techniques (Sueur et al., 2021) and might hold particular value for monitoring biodiversity (Burivalova et al., 2019). ‘Acoustic biodiversity’ has been suggested as an important contributor to wellbeing outcomes in natural environments (Ferraro et al., 2020, Sueur et al., 2021) but with current methods of soundscape analysis relying on complex computational techniques (Pijanowski et al., 2011), little is currently understood about how changes to an ecosystem’s soundscape might be experienced, or even noticed, by human non-specialists. Moreover, with increasing importance being placed on preserving pristine natural soundscapes (Buxton et al., 2017), how a change in acoustic composition might impact people’s motivations to conserve these environments remains unclear (Dumyahn and Pijanowski, 2011).

Concern for natural environments is, in part, influenced by socio-cultural factors (Gifford and Nilsson, 2014), reflecting the deep civilizational connections between nature and health (Ward Thompson, 2011). These interrelations are increasingly being explored through the use of creative prose, which is now often paired with natural sounds in commercially available relaxation tools (Headspace, 2021) and employed as a way to reconnect people with nature (National Trust, 2021). Although the presence of human voices can diminish the perceived tranquility of natural environments (Benfield et al., 2010), a narrow focus on these negative effects might obscure a possible synergy between nature and the use of spoken word in creative forms such as poetry. For example, in the right context, ‘culturally valued’ narratives can form positive compliments to a nature-based experience (Karmanov and Hamel, 2008) and poetry has demonstrated the potential to induce positive emotions in people (Obermeier et al., 2013). Recognizing their possible overlap, the unique format of *Forest 404* provided a platform to explore the interplay between nature-based poetry and natural soundscapes.

### 1.5 Possible moderating and mediating factors

To understand differential patterns in restorative potential and preservation motivation across changing soundscapes, we centered on a key moderator: lived experience. Memories of prior encounters with nature may be important for both increasing people’s wellbeing (Ratcliffe and Korpela, 2016) and stimulating pro-environmental behavior (Evans et al., 2018), with a reduction in nature-based experiences expected to have negative impacts on each of these outcomes (Kahn Jr and Kellert, 2002). Research has suggested the importance of lived experience in soundscape appraisals (Medvedev et al., 2015, Ratcliffe et al., 2016, Yang and Kang, 2005) and we sought to detect and quantify this moderating effect. Our experimental approach also made it possible to explore how psychological restoration might play a role in mediating pro-environmental behavior (Hartig et al., 2007): would participants demonstrate ‘human-first’ priorities by exhibiting higher motivations to preserve natural sounds if they thought they would be good for recovering depleted affective and cognitive resources?

Characteristics such as sex, age, and trait-based connection to nature can also impact responses to natural stimuli. For example, women and older people have reported greater feelings of calmness when listening to bird song (Hedblom et al., 2017), and women, younger people, and those more connected to nature have reported increased happiness and demonstrated a higher propensity for pro-environmental attitudes (Capaldi et al., 2014, Gifford and Nilsson, 2014, Whitburn et al., 2020, Zelezny et al., 2000). Detailed exploration of these individual differences was beyond the scope of the current paper but given their importance in previous studies, we also sought to account for their possible effects by including them as covariates in our analyses.

### 1.6 Research questions

Our research questions were intertwined with the narrative of *Forest 404*, inviting participants to make their own appraisals of varying natural soundscapes.

Research question #1 asked how the perceived restorative potential of a natural soundscape might be influenced by the sound types from which it is comprised. We anticipated that the presence of landscape elements such as flowing water (Yang and Kang, 2005) and audible fauna such as bird song (Ferraro et al., 2020) would be perceived to enhance restoration. However, we had little steer on how the addition of poetry might impact these appraisals. Similarly, how differing combinations of these sound types might impact restorative potential was highly exploratory.

Research question #2 probed the same areas as question #1, asking how preservation motivation might be influenced by soundscape composition. We expected the presence of natural sounds from biological sources to increase participants’ desires to preserve the soundscapes they heard. But once again, how the inclusion of poetry might affect these ratings, and how varying sound combinations would be perceived, was unclear given the lack of relevant prior research.

Research question #3 assessed how the patterns emerging from research questions #1 and #2 might be moderated by lived experience. Based on prior studies (Ratcliffe and Korpela, 2016) we expected positive memories of a soundscape to be associated with increases in restorative potential. The scale of this effect and whether it would be mirrored in ratings for preservation motivation, were novel areas of investigation.

Research question #4 was partly contingent on the outcomes of questions 1–3; if soundscape composition and lived experience were associated with appraisals of restorative potential and preservation motivation, might restorative potential mediate preservation motivation? We suspected participants may be more motivated to preserve soundscapes they believed would provide therapeutic outcomes (Hartig et al., 2007), but the scant literature in this area of environmental sensing meant we could not hypothesize about the magnitude and consistency of this relationship.

Across each of these research questions we also included sex, age, and connectedness to nature as covariates.

## 2 Methods

Our experimental approach presented respondents with three natural soundscapes, randomly selected and ordered, and asked them to appraise the sounds they heard according to several dependent measures. To facilitate a between-participant design and prevent possible ordering effects, we only considered data from respondents’ first sound in the analyses presented here.

### 2.1 Participants

We hoped to collect a minimum of 50 responses per stimulus (50 × 36 conditions = 1,800 in total) based on previous soundscape studies where between 30 and 50 participants per condition have been sufficient to detect inter-stimulus differences in restoration and affect (Alvarsson et al., 2010, Medvedev et al., 2015, Payne, 2013). Participants were recruited via a call-to-action in the credits of each *Forest 404* episode. Those who were interested in taking part followed an online link to the experiment. Participation was open for seven months, from 4th April to 31st October 2019. Most respondents (94%) took part within the first 3 months of study recruitment. No remuneration was provided in return for participation and respondents were informed that the study aimed to improve “*Understanding of people’s feelings about nature-based sounds and poetry*”. No additional information about hypotheses and methods was provided.

7,596 participants completed the experiment, four times the required sample size. Only finalized responses were recorded, we do not know how many people started but did not complete the experiment. Modal age range was 46–55, 30% of our sample was aged 35 or under, 63% were female, 35% male, 0.7% identified as ‘Another sex or gender’. Most participants (87%) were UK residents, we did not record the location of international respondents. Two-thirds of participants (67%) reported visiting nature at least once in the last week and mean self-reported connectedness to nature was 7.02 on a 10-point scale (Table S1, Appendix A). Compared to UK averages (ONS, 2019, Richardson et al., 2019, White et al., 2017), our sample was slightly biased towards females, those who were older, and people more interested in the natural world, but not excessively so compared with similar studies (Richardson and McEwan, 2018).

### 2.2 Experimental design

We employed the acoustic categories ‘geophony’, ‘biophony’, and ‘anthrophony’ used in soundscape ecology (Pijanowski et al., 2011) but renamed our sound types to provide a succinct labelling structure. Abiotic sounds (A) represented the aural signature of the landscape, such as waves breaking and water flowing; whilst Biotic sounds (B) stemmed from fauna within an environment, including the sounds of birds, livestock, and, in our underwater biome, whale song. Our ‘Culturally valued’ poems (C) each depicted their respective environment and, to enhance integration with the wider series, were read by *Forest 404* actor, Pippa Haywood.

**Fig. 1:**
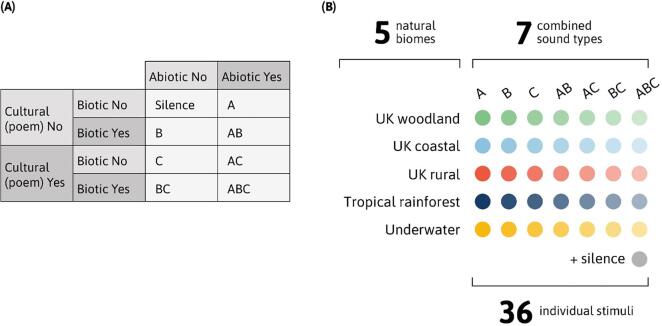
Arrangement of stimuli in study design. (A) Factorial arrangement of sound types within a single biome. (B) Total stimuli arising from factorial design applied across five biomes. Example stimulus: Using this structure, the ABC stimulus in our tropical rainforest biome was created by combining (A) the abiotic sound of rain falling on leaves, with (B) the biotic sounds of indigenous New Guinea birds, and (C) a spoken extract from ‘Savage Grace: A Journey in Wildness’ by Jay Griffiths (read by *Forest 404* actor, Pippa Haywood).

To create soundscapes of differing composition and increasing complexity, stimuli were arranged in the 2 × 2 × 2 factorial design (A = Yes/No; B = Yes/No; C = Yes/No) depicted in Fig. 1A. To broaden the applicability of the study beyond responses to a single environment, this design was repeated across five biomes: UK woodland; UK coastal; UK pastoral; tropical rainforest; and underwater. The three UK-based environments were chosen because they reflected common Eurasian soundscapes likely to be familiar to much of the *Forest 404* listenership, and therefore elicit mixed valence memories. The fourth biome, a tropical rainforest, was more exotic in origin and closely resembled the soundscape Pan encountered in the *Forest 404* series. The final biome, an underwater ocean soundscape, was selected because of its frequent use in relaxation settings (Lin et al., 2011). This design resulted in a total of 36 conditions (Fig. 1B) and aimed to reduce the chance that results might reflect reactions to a specific sound, instead revealing more generalizable patterns across contexts. Since the focus of the analyses reported here was on changing soundscape composition, responses to sound types were collapsed across biomes resulting in eight conditions: seven soundscapes and our silent control.

### 2.3 Experimental stimuli

The abiotic and biotic sounds chosen to represent each of our auditory biomes were intended to be broadly calming. Drawing on archival recordings at the BBC, specific sounds were selected based on preferences already established in the literature (Buxton et al., 2021, Carles et al., 1999, Hedblom et al., 2014, Ratcliffe et al., 2016, Yang and Kang, 2005). Nature-based poems (‘C’ sounds) were selected to match their corresponding biome with significant input from producers at BBC Radio 4′s ‘Poetry Please’ program. For example, ‘Dover Beach’ by Matthew Arnold was chosen for the coastal soundscape, whilst ‘Woods’ by Wendell Berry was paired with sounds in the woodland biome. Rights to all sounds were obtained by the BBC, with explicit consent received from artists where necessary. The specific list of sounds is shown in Table 1. Based on previous studies (Hedblom et al., 2014) and following extensive piloting, stimulus duration was set at 40 s.

**Table 1:** Description of specific sounds used in experimental conditions. As shown in Fig. 1, sounds were arranged in a 2x2x2 design within biomes. For example, an AB sound in the UK coastal biome would feature both ‘calm waves lapping on the beach’ and ‘oystercatchers chirping’; an ABC sound would add the poem ‘Dover Beach’ by Matthew Arnold to this mix.

**Biome**	**Sound A**	**Sound B**	**Sound C**
UK woodland	Swirling wind with leaves rustling	Woodland birdsong with blackbird	‘Woods’ by Wendell Berry
UK coastal	Calm waves lapping on the beach	Oystercatchers chirping	‘Dover beach’ by Matthew Arnold
UK rural	Gentle stream flowing	Hedgerow birds with distant sheep bleating	‘Spring’ by Gerard Manley Hopkins
Tropical rainforest	Heavy rain with distant thunder	Various bird calls from the New Guinea rainforest	Extract from ‘Savage Grace: A Journey in Wildness’ by Jay Griffiths
Underwater	Underwater waves crashing and sloshing	Humpback whale calls	‘Underwater’ by Michael Schmidt

### 2.4 Experimental instrument

Ethical approval for the present study was granted by the University of Bristol Faculty of Arts Committee for Research Ethics, Ref: 76582.

The experiment was hosted online via The Open University’s nQuire platform (The Open University, 2021). Following a brief introduction, participants had to provide informed consent before taking part. They then listened to a test sound to ensure their speakers were working and set to a comfortable volume. Respondents then read a stress-inducing vignette. This approach was used due to the online nature of the experiment, which did not allow real stress inducement and measurement of recovery. The narrative was adapted in accordance with previous studies (Ratcliffe et al., 2016, Staats and Hartig, 2004) and asked participants to imagine a situation in a typical urban setting that had led them to feel stressed and cognitively fatigued. To enhance immersion in the story, participants were asked to listen to a busy city soundscape, featuring traffic and construction noise, while they read the vignette. Given wide potential heterogeneity in aural experiences before taking part in the experiment, the vignette and soundscape were designed to harmonize the immediate experiences of all participants (to be unpleasant and mildly stressful) before exposure to the experimental conditions.

Participants then listened to one of our nature-based soundscapes, randomly chosen from the pool of 36. They were instructed to listen to the sound in full first, with their eyes closed if possible. When the sound had finished playing, they were asked to scroll down and respond to a series of questions (detailed in section 2.5). Participants could play the sound again or answer questions before having heard all of it. We could not record the time spent listening to each sound. After their first stimulus, participants repeated this process for another two soundscapes, randomly chosen and ordered by the nQuire software. The 40-second duration of stimuli and use of relatively few response scales aimed to keep average completion time below ten minutes (determined from pilot testing), maintain participant interest, and encourage full completion. The experiment ended with a series of demographic items. An overview of the experimental procedure is available in Appendix A and full wording, including an example of the user-interface, is available on the Open Science Framework (https://doi.org/10.17605/OSF.IO/P3GTY). As previously mentioned, to maximize relevance to the initial vignette and to avoid possible ordering effects, we only consider data from respondents’ first sound in the analyses presented here (between 199 and 218 participants per soundscape and ∼1000 responses per condition collapsed across biomes).

### 2.5 Measures

The need for a short online experiment precluded the use of multi-item psychometric measures. In line with other creative data gathering exercises (Richardson and McEwan, 2018), short-version scales and single item metrics were thus used for several dependent variables. Given the reach of our unique recruitment opportunity, the experiment included a wide variety of questions. Measures not included in the present study captured appraisals of valence, arousal, and generalized preference.

#### 2.5.1 Perceived restorative potential

Our composite measure of therapeutic potential comprised three items each measured on a ten-point scale: perceived restorative potential, fascination, and being away. The single item measure of restorative potential used wording adapted from several other studies (Herzog et al., 2003, Ratcliffe et al., 2016) and asked “*Thinking about your stressful scenario, to what extent do you think listening to this sound would help you recover and feel better in that moment?*”

Items for ‘fascination’ and ‘being away’ – two core components of a restorative experience (Kaplan, 1995) – were adapted from several permutations which exist in the current literature (Hartig et al., 1997, Payne, 2013). The fascination item asked “*To what extent do you agree with this statement? ‘Listening to this soundscape is fascinating; it holds my interest and awakens my curiosity.’*” The ‘being away’ item asked “*To what extent do you agree with this statement? ‘Listening to this soundscape allows me to feel far away from everyday thoughts and concerns.*’”

The personal pronoun (“my” or “me”) was included to ensure respondents were considering the restorative potential for themselves, rather than via a more objective perspective (Payne and Guastavino, 2018). Each item was rated on a 10-point scale, from “*Not at all*” (1) to “*Completely*” (10). Inter-item correlations for these measures were high (0.64 < r < 0.75) and they were subsequently collapsed into a combined measure of perceived restorative potential (α = 0.88).

#### 2.5.2 Preservation motivation

Preservation motivation was measured using a novel item designed to prompt a hypothetical decision to ‘keep’ or ‘delete’ a soundscape, with the latter action removing the sound from recorded history. It required participants to appraise the severity of irreversible loss of their sound, for themselves and wider society. It was deliberately analogous to the choices Pan faces in *Forest 404*, both in the data archive and when she is forced to trade her soundscapes in place of financial payment (*Episode two: The Fumetown Priest*). Although links to the experiment were only available via the podcast, we could not rule out that some participants may not have listened to the drama. The experiment information sheet thus provided background on Pan’s role and the question was worded to make sense to those who could have found the experiment via alternative routes. Specifically, it asked “*Imagine you are Pan from the Forest 404 podcast. You are working in the data library and this is the file you have just been asked to process. What do you think you would do with this sound?*” Responses were captured on a 10-point scale from “*Definitely delete*” (1) to “*Definitely keep*” (10). A higher rating indicated a greater desire to keep rather than discard the stimulus.

#### 2.5.3 Memories

Following previous research demonstrating the importance of lived experience in soundscape appraisals (Dumyahn and Pijanowski, 2011, Medvedev et al., 2015) we asked if participants had memories triggered by the soundscape they were listening to, and if so, to state the valence of these memories. The question was “*Do you have any memories associated with this kind of sound? If so, are they mostly positive, negative or mixed?*” Participants could answer with one of the following responses: *No memories; Mostly positive memories; Mostly negative memories; A mix of positive and negative memories*.

#### 2.5.4 Individual difference covariates

Respondents were asked to state their sex and could identify as: *Female; Male; Another sex or gender*. ‘Another sex or gender’ was included as a factor level in all analyses, but low prevalence (0.7%) in our sample precluded the statistical power necessary to identify significant trends and this group is subsequently omitted from descriptions of findings.

Age was captured in groups spanning ten-year bands (e.g. 36–45). All age groups were included as covariates in analyses with consistent positive associations for those aged 36 and over. To simplify reporting, and based on observed patterns in the different groups, age was collapsed into two categories, with those aged between 18 and 35 in one group, and those aged 36 and over in the second group.

To reduce participant burden from longer scales (Richardson et al., 2019), connectedness to nature was measured using a single item adapted from the Inclusion of Nature in Self scale (Schultz, 2002). Participants were asked “*Thinking about your place in the world, to what extent do you feel 'part of nature'?*” Responses were registered on a 10-point scale from “*Not at all*” (1) to “*Completely*” (10).

The full list of demographic items captured in this study is presented in the ‘Demographic questions’ section of Appendix A.

### 2.6 Statistical analysis

**Fig. 2:**
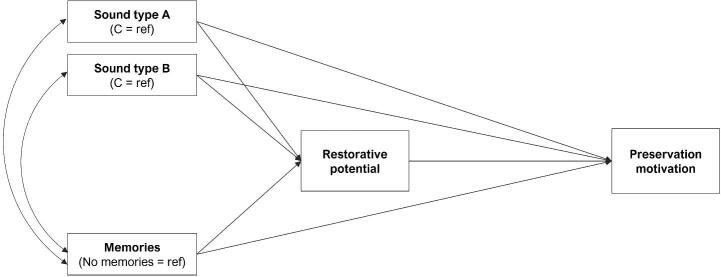
Mediation pathways. The planned mediation model used to explore research question 4, with sound type (A, B, C) and memories (any vs none) as predictors, preservation motivation as outcome, and restorative potential as mediator. Covariances of residuals depicted by double headed arrows.

All analyses were conducted using the statistical software R (R Core Team, 2021). To answer research questions 1–3, outcomes were analyzed using a between-subjects ordinary least squares linear regression, with main effects for all factors included. To explore research question 4, a mediation analysis was conducted using the structural equation modelling package ‘Lavaan’ (Rosseel, 2012). We constructed a simplified path model (Hayes, 2017) with sound type (A, B, C) and memories (any vs none) as predictors, preservation motivation as outcome, and perceived restorative potential as mediator, as depicted in Fig. 2. The model ran 1,000 resamples. Sex, age, and connectedness to nature were included as covariates in all models. Since all dependent variables used the same ten-point scales, we present the unstandardized coefficients in each figure to aid comparisons between analyses. We also initially report mean appraisals of sounds collapsed according to biome, with differences between groups assessed via a one-way ANOVA. Related post hoc tests have been Bonferroni adjusted. Although briefly presented in section 3, further expansion of these analyses is beyond the scope of the current research. Full tabular outputs are presented in Appendix A. Data are available on the Open Science Framework (https://osf.io/p3gty).

## 3 Results

### 3.1 Preliminary results across biomes

**Fig. 3:**
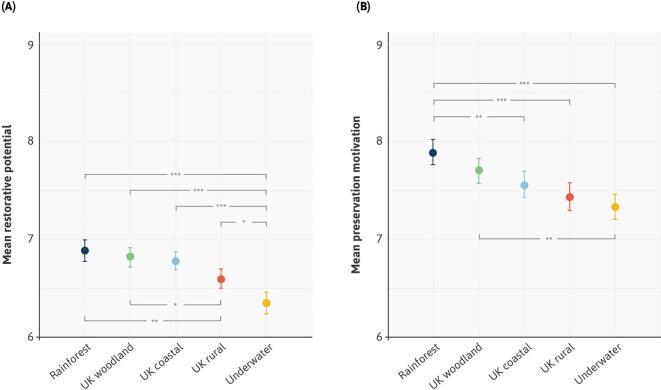
Soundscape appraisals according to biome. Mean scores for (A) perceived restorative potential and (B) preservation motivation, for all sound types (excluding silence) collapsed according to biome. Asterisks highlight significant differences, * denotes p < 0.05, ** denotes p < 0.01, and *** denotes p < 0.001. Pairwise comparisons have been Bonferroni corrected. Confidence intervals (95%) are also displayed.

Aggregating responses for all sound types, appraisals for our key metrics varied by less than a scale point across the five biomes (Fig. 3). However, small but significant differences existed for both perceived restorative potential (F (4, 7249) = 16.38, *P* < 0.001) and preservation motivation (F (4, 7289) = 9.54, *P* < 0.001). Broadly speaking, soundscapes from the tropical rainforest, the signature soundscape of the *Forest 404* series, were rated highest for both restorative potential (Fig. 3A) and preservation motivation (Fig. 3B). Sounds from our underwater biome were rated the least positively on both outcomes. Appraisals for sound types were highly similar across environments (see Fig. S1 and S2 in Appendix A), so for the remainder of the paper we have collapsed analyses across biomes to focus on our primary research questions.

### 3.2 Hierarchies between soundscapes

**Fig. 4:**
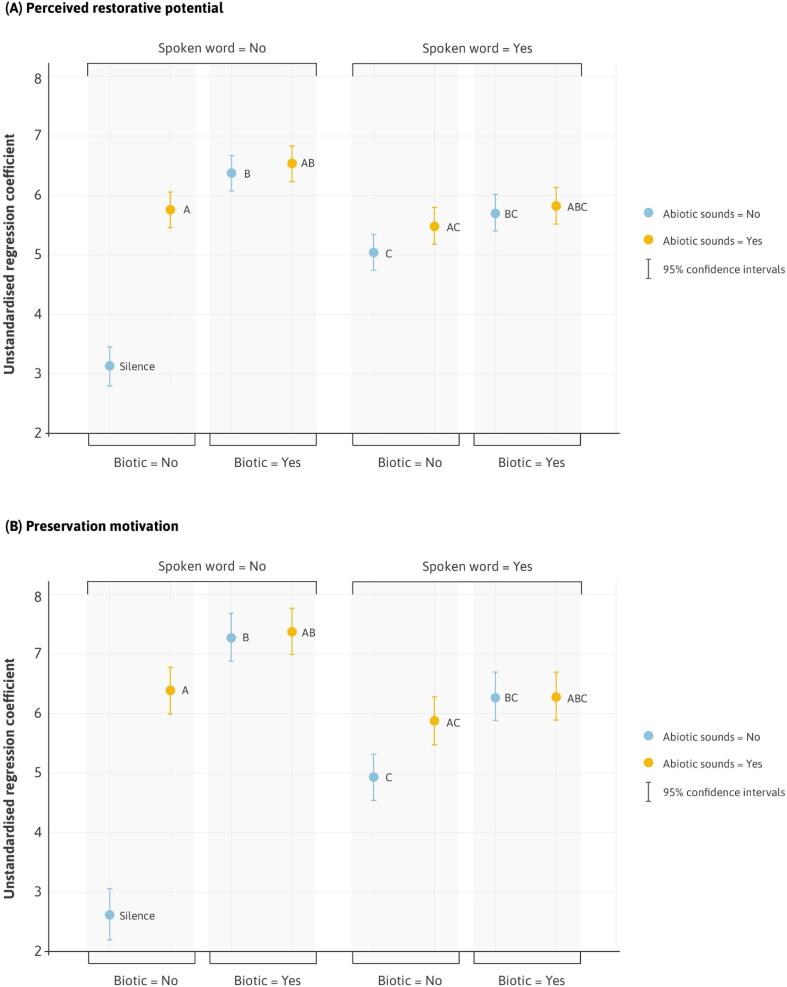
Delineating according to sound types. Unstandardised coefficients for (A) perceived restorative potential and (B) preservation motivation. The y-axis represents a range that captures all the variation in responses. To aid visualization, regression coefficients have been added to the intercept (Silence). Confidence intervals (95%) are also displayed.

Starting with research question #1, we began with an examination of variations in the perceived restorative potential of our stimuli (Fig. 4A, see Table S2 and S3 in Appendix A for tabular outputs). Compared to the silent control condition, soundscapes that combined abiotic and biotic elements (‘AB’) were perceived as most restorative (*B* = 3.41, SE = 0.15, t(7133) = 18.63, *P* < 0.001). Biotic sounds alone (‘B’) were rated as significantly more restorative than silence (*B* = 3.26, SE = 0.15, t(7133) = 17.02, *P* < 0.001) with no significant difference between these and our combined AB sounds (*B* = − 0.16, SE = 0.09, t(7133) = -1.79, *P* = 0.074). Abiotic sounds (‘A’) were rated as more restorative than silence (*B* = 2.63, SE = 0.15, t(7133) = 17.02, *P* < 0.001), but significantly lower than our combined AB sounds (*B* = − 0.78, SE = 0.09, t(7133) = -8.92, *P* < 0.001). Put simply, our most acoustically rich natural soundscapes – containing both abiotic and biotic sounds – were the most restorative. When we removed biotic sounds (such as birdsong) from these soundscapes, to leave only the abiotic sounds of the landscape (such as flowing water), we observed a clear reduction in perceived restorative potential.

On their own, our ‘culturally valued’ poems (‘C’) were rated as more restorative than silence (*B* = 1.92, SE = 0.15, t(7133) = 21.05, *P* < 0.001), but significantly less so than the nature-only sound types described above (Table S4). However, adding nature-based sounds to our poems had a consistent positive effect. For example, the inclusion of abiotic and biotic sounds (‘ABC’) significantly increased ratings of restorative potential compared to poetry alone (*B* = 0.78, SE = 0.09, t(7133) = 8.85, *P* < 0.001).

Addressing research question #2, patterns in participant motivations to preserve their soundscapes were very similar (Fig. 4B, Tables S5-7 in Appendix A for tabular outputs). Compared to silence, combined abiotic and biotic soundscapes (‘AB’) had the highest preservation ratings (*B* = 4.81, SE = 0.20, t(7173) = 23.80, *P* < 0.001). Again, biotic sounds alone (‘B’) were no less likely to be preserved than AB sounds (*B* = -0.10, SE = 0.11, t(7173) = -0.90, *P* = 0.37). However, removing biotic sounds to leave only abiotic elements (‘A’), significantly decreased preservation motivation (*B* = -0.99, SE = 0.11, t(7173) = -8.65, *P* < 0.001). Poetry was more likely to be preserved than silence (*B* = 2.33, SE = 0.20, t(7173) = 11.51, *P* < 0.001), but less so than our nature-only sounds. Once again, combining nature-based sounds with poetry had a positive effect. For example, the addition of abiotic and biotic sounds (‘ABC’) significantly increased preservation motivation compared to poetry alone (*B* = 1.37, SE = 0.12, t(7133) = 11.87, *P* < 0.001).

With respect to our covariates, we observed a positive association between perceived restorative potential and connection to nature across all sound types; participants who felt more connected to the natural world rated their sounds as more restorative (*B* = 0.13, SE = 0.01, t(7133) = 11.19, *P* < 0.001). We detected no relationship with age or sex for perceived restorative potential. However, for preservation motivation greater individual differences existed. Females exhibited higher preservation motivation ratings than males (*B* = 0.19, SE = 0.06, t(7173) = 3.00, *P* = 0.003); and participants aged 36 and over returned higher average ratings than those aged between 18 and 35 (*B* = 0.24, SE = 0.07, t(7173) = 3.62, *P* < 0.001). Those reporting higher connectedness to nature were also more likely to want to keep the soundscapes they listened to (*B* = 0.13, SE = 0.02, t(7173) = 8.68, *P* < 0.001).

### 3.3 The moderating role of memories

Next, we considered research question #3 and explored how participants’ memories might moderate responses to our stimuli. The format of our memory-based question prevented us from interpreting memories for our combined soundscapes (we could not determine which component the memory related to), so for these analyses we focused on single component soundscapes only (A, B, or C). The silent condition was also not considered here.

**Fig. 5:**
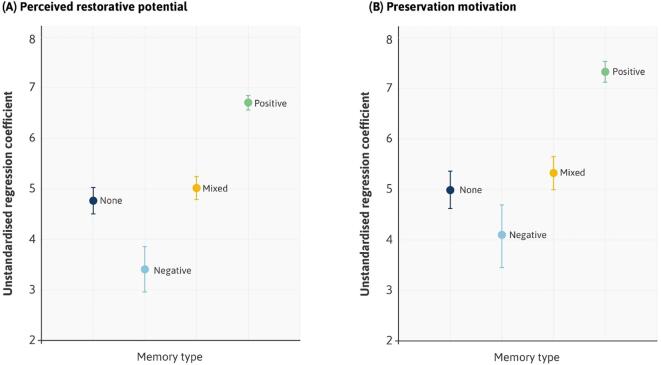
Soundscape ratings grouped by participant memories. The relationships between memory type and (A) perceived restorative potential and (B) preservation motivation, across abiotic, biotic and poetry-based sound types collapsed together. The y-axis represents a range that captures all variation in responses. To aid visualization, regression coefficients have been added to the intercept (memories = none). Confidence intervals (95%) are also displayed.

Collapsing our soundscapes together, we observed a significant main effect of memory type on perceived restorative potential (Fig. 5A, Table S8). Compared to those with no prior memories of their sounds, negative memories had a significant detrimental effect on ratings of restorative potential (*B* = -1.36, SE = 0.22, t(2987) = -6.11, *P* < 0.001). Positive memories exerted the opposite effect, increasing ratings by nearly 2 scale points (*B* = 1.94, SE = 0.08, t(2987) = 25.02, *P* < 0.001). Mixed memories led to a small yet still significant increase (*B* = 0.25, SE = 0.12, t(2987) = 2.09, *P* = 0.037).

The same pattern existed in participants’ motivation to preserve their sounds (Fig. 5B, Table S9). Compared to those with no memories, negative memories reduced ratings (*B* = -0.90, SE = 0.31, t(3002) = -2.89*P* = 0.004) whilst positive memories substantially increased them (*B* = 2.33, SE = 0.11, t(3002) = 21.44, *P* < 0.001). Mixed memories led to a slight increase in preservation motivation (*B* = 0.34, SE = 0.17, t(3002) = 2.03, *P* = 0.043).

Once again, females (*B* = 0.37, SE = 0.10, t(3002) = 3.83, *P* < 0.001), those aged 36 and over (*B* = 0.33, SE = 0.10, t(3002) = 3.21, *P* = 0.001), and those who were more connected to nature (*B* = 0.08, SE = 0.02, t(3002) = 3.54, *P* < 0.001) had higher preservation motivation ratings. Only connection to nature was a significant covariate for restorative potential (*B* = 0.08, SE = 0.02, t(2987) = 4.73, *P* < 0.001).

### 3.4 The effects of memories on individual sound types

We might reasonably assume that participants without memories of our sounds had interacted less with natural environments over their lives than those with memories, regardless of whether those memories were positive or negative. To explore the possible impact of this extinction of experience on our individual sound types (Soga and Gaston, 2016), we collapsed our negative, mixed, and positive memory categories together to form a single group of participants with memories of the sounds they heard (n = 2244), and compared this subset to those without (n = 808).

**Fig. 6:**
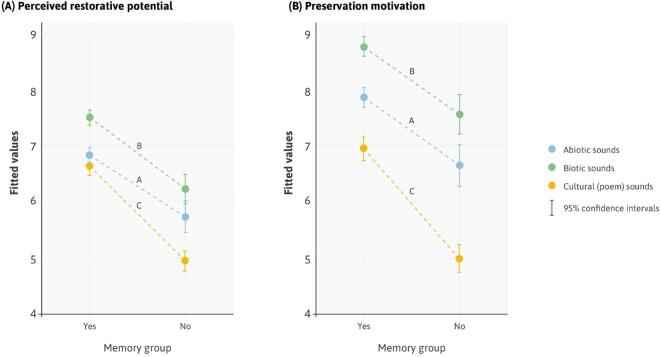
The effect of memories on specific sound types. Fitted model values for A, B, C sound types and memory group for (A) perceived restorative potential and (B) preservation motivation.

Fitting estimated marginal means to our model, Fig. 6 depicts a significant main effect of memories. Each of our sound types received higher ratings of perceived restorative potential (Fig. 6A, Tables S10 and S11) from those who had memories triggered by the experience compared to those who did not (*B* = 1.11, SE = 0.16, t(2985) = 7.11, *P* < 0.001). The pattern for preservation motivation was similar yet even more pronounced (Fig. 6B, Tables S12 and S13). Those reporting memories were much more likely to preserve each sound type than those with no memories (*B* = 1.21, SE = 0.21, t(3000) = 5.84, *P* < 0.001). Significant interaction terms also suggested that for both perceived restorative potential (*B* = 0.58, SE = 0.20, t(2985) = 2.92, *P* = 0.003) and preservation motivation (*B* = 0.74, SE = 0.26, t(3000) = 2.83, *P* = 0.005), a lack of memories had a disproportionately larger impact on responses to poetry (C) than either abiotic (A) or biotic (B) sounds, as reflected in the steeper downward sloping lines in Fig. 6.

### 3.5 Restorative potential as a mediator of preservation motivation

**Fig. 7:**
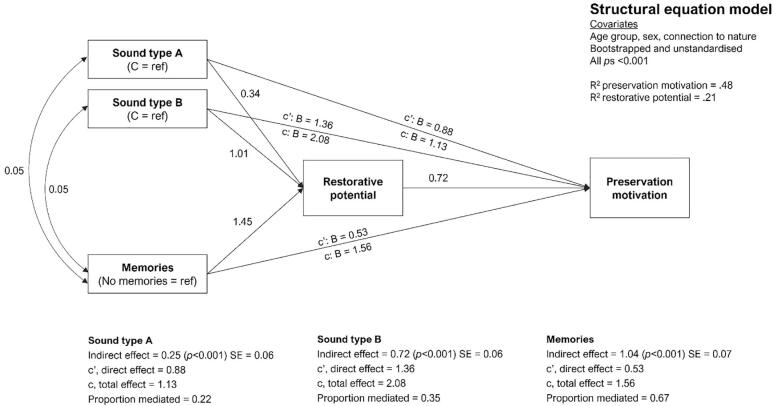
Mediation model. Structural equation model with sound type (A, B, C) and memories (any vs none) as predictors, preservation motivation as outcome, and restorative potential as mediator. Covariances depicted by double-headed arrows. Tabular outputs can be found in Table S15, Appendix A.

The similarity between patterns for perceived restorative potential and preservation motivation described above reflects their strong association (r = 0.64, *P* < 0.01, Table S14) and is indicative of potential mediation; the reason why participants may want to ‘keep’ certain soundscapes from being deleted may be because they present the opportunity for psychological restoration (Hartig et al., 2007), rather than holding intrinsic value in their own right (Dearborn and Kark, 2010). To address research question #4, we therefore examined the extent to which the restorative potential of our soundscapes might mediate preservation motivation, and the role memories may play in this relationship. We constructed a simplified path model (Hayes, 2017) with sound type (A, B, C) and memories (any vs none) as predictors, preservation motivation as outcome, and perceived restorative potential as mediator. Results indicated that restorative potential partially mediated the effects of sound type and memories on preservation motivation (Fig. 7). The bootstrapped (samples = 1000) and unadjusted indirect effects via restorative potential accounted for 22% and 35% of the total effects of abiotic and biotic sounds on preservation motivation, respectively (compared to poetry, the reference category). The unadjusted, indirect effect of memories via restorative potential on preservation motivation was 67% (*B* = 1.04, SE = 0.07, *P* < 0.001) of the total effect. In other words, a fifth of the effect of abiotic sounds, a third of the effect of biotic sounds, and two thirds of the effects of lived experience on participant decisions to preserve their stimuli were mediated by the restorative potential they might offer (see Fig. 7 and Table S15).

## 4 Discussion

The potential for the arts and sciences to co-develop novel methods that engage people in ecological issues is receiving increasing attention (Sommer et al., 2019). The *Forest 404* podcast embraced these principles, inviting listeners to imagine themselves as the series’ protagonist, who exhibited an intrinsic positive reaction to natural sounds – even though she had never experienced them before. Did our participants’ responses support the assumptions underpinning the *Forest 404* narrative?

### 4.1 Findings

Results demonstrate that nature-based soundscapes were valued differently according to their composition. Participants were more motivated to preserve sounds that featured biotic elements, such as bird song or pastoral fauna, and believed they would find these soundscapes to be most restorative in times of stress and cognitive fatigue. When we removed biotic sound sources to simulate the kind of impoverished environmental experience portrayed in *Forest 404*, perceived restorative potential and preservation motivation both fell. To put it another way, as the soundscape appeared to reflect a decline in environmental quality, participants’ sense that the environment would offer psychological benefits also fell and their motivation to protect those environments appeared to follow suit.

Crucially, our findings demonstrate that when it comes to nature, memories matter. Without memories of the soundscapes they heard, participants were significantly less likely to find them restorative and were less motivated to preserve them. These findings challenge the *Forest 404* narrative and suggest reduced environmental experience may have a significant effect on responses to nature-based stimuli. Moreover, our results highlight the potential importance of psychological restoration in appraisals of natural capital. Two-thirds of the total effect of memories on participant motivations to preserve natural sounds was mediated by the restorative potential they might offer. Interactions with nature can foster pro-environmental attitudes (Alcock et al., 2020) and our results suggest that psychological restoration could be an important pathway through which this mechanism operates.

When listened to on its own, nature-inspired poetry received lower ratings of restorative potential and preservation motivation than natural soundscapes. The addition of abiotic and biotic sounds increased these ratings, suggesting context-specific natural soundscapes might enhance both the evaluation and therapeutic potential of poetry. However, this relationship can also be viewed more pessimistically; adding poetry to our nature-based sounds led to a significant drop in positive appraisals compared to natural sounds alone.

The effects of age and sex were relatively consistent across our results. Females and those aged 36 and over were, on average, more likely to preserve their soundscapes compared to males and younger people. Participants who felt more connected to nature also exhibited a higher tendency to want to ‘keep’ their soundscapes. In contrast to the cynical motivations described above, these patterns provide support for the effects of ‘nature-first’ conservation priorities among these groups (Dearborn and Kark, 2010), and underline the positive links between connectedness to nature and environmental behavior (Whitburn et al., 2020). Consistent with previous findings (Capaldi et al., 2014), increased ratings of restorative potential were also positively associated with connection to nature.

### 4.2 Limitations

Despite the large size and diversity of our study population, some limitations must also be acknowledged. Our sample was self-selecting, and participants tended to be older, more connected to nature, and more likely to be female than UK averages. Recruitment to the experiment was almost exclusively via the *Forest 404* series. We do not know how much of the podcast participants had listened to, nor the degree to which its narrative might have influenced their responses. Our experimental design simulated ecosystem degradation by removing all wildlife sounds from the acoustic environment. This kind of severe change in soundscape composition has previously been considered a portent of environmental damage, embodied by the notion of a ‘silent spring’ (Carson, 1962). Yet real biodiversity loss tends to happen at a more gradual rate, and most species do not contribute to the soundscape. Future work might look at the impacts of more nuanced changes, particularly with respect to the impact of ‘shifting baselines’ and the notion that people readily adapt to slow shifts in reference states (Pauly, 1995).

To reduce participant burden, we used soundscapes that were 40-seconds long. We do not know how outcomes may have varied for longer exposures, particularly for our poem-based sounds. Our preservation motivation question asked respondents to imagine a situation in which they had to ‘keep’ or ‘delete’ the sounds they were hearing. Since this behavior was hypothetical and did not have demonstrable consequences, we must be careful when drawing parallels with actions in real-world situations. Our measure of lived experience captured a general sense of participant memories, but we could not determine at what point in the life course these memories occurred or whether they were truly autobiographical. Respondents reported having memories of our more exotic soundscapes, suggesting that responses might also reflect associations assembled from a broad mix of experiences, including natural history programming. The diversity of what people consider to be ‘lived experiences’ of nature could be a beneficial focus of future research (Ballouard et al., 2011).

### 4.3 Implications

The restorative potential of varying acoustic sources has often been considered interchangeably under the broad banner of ‘natural sounds’ (Alvarsson et al., 2010, Gould van Praag et al., 2017). Yet emerging evidence suggests these approaches may have overlooked differential contributions of specific sound types (Buxton et al., 2021). Through the systematic manipulation of soundscapes from five contrasting biomes, our results suggest that significant heterogeneity exists in the appraisal of environmental stimuli already broadly defined as therapeutic, and reveal nuance in the notion of ‘tranquil space’ (Pheasant et al., 2010).

We find that abiotic sounds explored by other studies, such as wind and flowing water (Ratcliffe, 2021), are significantly enhanced by the addition of sounds from biotic sources, such as bird songs and calls. Acoustic ecologists have recently begun to consider ‘biophony’ as a vital marker of ecosystem health (Pijanowski et al., 2011) and our findings suggest that non-specialists may also detect when audible components of biodiversity are missing. These outcomes are particularly striking because participants were not making a comparison between soundscapes with and without wildlife (due to our between-participant design), yet they reacted differently when it was missing. The presence of bird song might form an important contributor to wellbeing outcomes in natural settings (Ferraro et al., 2020) and we demonstrate how this trend may extend to a wider range of acoustic biodiversity (Sueur et al., 2021).

How might these findings inform practice? One pathway could be through the inclusion of specific natural soundscapes – and their subsequent restorative potential – in psychological ecosystem services (Bratman et al., 2019), recognizing biodiverse soundscapes as natural capital and incorporating them into existing models designed to map and quantify these services (Paulin et al., 2020). Our results might also feed into the design of restorative public spaces (Yang and Kang, 2005) by promoting efforts to protect and create habitats that feature wildlife and its associated aural markers (Levenhagen et al., 2021). Soundscape appraisals can play a considerable role in determining landscape preferences (Gan et al., 2014) yet acoustic environments are in constant temporal flux (Matsinos et al., 2008). Sonic signatures such as breaking waves and falling rain can vary with sporadic shifts in the weather, whilst the sounds of bird song and other fauna are likely to follow diurnal and seasonal patterns. Our data provide evidence to suggest these variations might also be considered alongside visual ephemeral features in landscape assessments (Brassley, 1998).

Supporting early theorizing (Kaplan, 1995, Ulrich, 1983) and more recent extensions (Ratcliffe and Korpela, 2016), our findings further validate the importance of top-down processes such as memories in environmental appraisals. Although more often explored in qualitative studies (Conradson, 2005), the relatively large effects of prior memories on our results suggests that these and other top-down processes should be more prominent in future quantitative soundscape investigations. Moreover, the effects of memories extended to participant motivations to preserve their sounds. Engagement with the natural world in early life can lead to positive environmental attitudes later on (Nancy and Kristi, 2006) and our results provide further support for this effect.

Viewed in reverse, this relationship paints a stark picture of the impacts stemming from the potential extinction of nature-based experiences. People who had no previous memories of their soundscapes were less likely to believe they could gain wellbeing benefits from listening to them and were less motivated to protect them. *Forest 404* implicitly asked audiences “*Can you feel loss for something you have never known?*” The profound effects of memories in our results suggest the answer to this question might, worryingly, be “*no*”. If societal trends continue to demonstrate a disconnection of populations from the natural world (Hunt et al., 2016), a negative feedback loop for both wellbeing and environmental preservation may ensue (Soga and Gaston, 2018) – although also see (Novotný et al., 2020, Oh et al., 2020).

Questions have been raised about the pathways through which nature experience might impact the valuing of natural environments (Neuteleers and Deliège, 2019). We present evidence to suggest that appraisals of therapeutic potential could be a viable mediating mechanism in this relationship. This outcome is consistent with an ‘egoistic’ motivation for environmental protection, in which a person makes decisions based on outcomes likely to affect them personally (Stern and Dietz, 1994). Repercussions for the extinction of experience are once again writ large, but these findings could also have implications for conservation messaging. The use of shock and fear to motivate behaviors which address trends such as biodiversity loss is increasingly ineffective in a world where people have a limited ‘pool of worry’ (White et al., 2020). By making it clear that individual wellbeing could stand to benefit from nature protection, a reciprocal relationship might motivate people to preserve natural ecosystems (Soga and Gaston, 2016).

Existing research suggests that poetry can contribute to a range of positive wellbeing outcomes (Obermeier et al., 2013) and we find, for nature poetry at least, that the addition of natural sounds may enhance these effects. These outcomes might be particularly useful for those aiming to connect people to the natural world through creative endeavors (National Trust, 2021), or harness the restorative power of literature and nature through bibliotherapy (McKenna et al., 2010) and emerging digital interventions (Headspace, 2021).

### 4.4 Conclusions

As global environmental changes continue to alter acoustic experiences, our results contribute to efforts to improve understanding of how soundscapes might impact human wellbeing and behavior (Smith and Pijanowski, 2014). They also take on new meaning following responses to the COVID-19 pandemic. In western societies at least, strict lockdowns re-focused attention on the relationships between nature and health (BBC News, 2020). As reductions in anthropogenic noise resulted in a quieting of both urban and rural environments, natural sounds were highlighted as a crucial component of the aural experience (Derryberry et al., 2020). With many people confined to their homes for prolonged periods, an interest in how digitally-mediated nature experiences might shape wellbeing also entered public discourse, reigniting debates surrounding the potential value of ‘virtual nature’ (Depledge et al., 2011). How sound and nature-based narratives might fit into this conversation could be an important focus of future work.

This study represents just one part of the BBC *Forest 404* project, a collaborative and award-winning public engagement initiative. This transdisciplinary series merged fictional, factual, immersive, and experimental elements, and encouraged audiences to contribute to scientific understanding. *Forest 404* demonstrated the power of creative alliances and provides a further exemplar for partnerships aiming to develop novel methods that enrich engagement in, and understanding of, environmental futures.

## Funding

Support for this research was provided by the Wellcome Centre for Cultures and Environments of Health, grant number 203109/Z/16/Z (AS). The *Forest 404* production was part-funded by the Arts and Humanities Research Council, grant number AH/P504622/1 (PC).

## Declaration of Competing Interest

The authors declare that they have no known competing financial interests or personal relationships that could have appeared to influence the work reported in this paper.
