# Supplementary material for: Forest 404: Using a BBC drama series to explore the impact of nature’s changing soundscapes on human wellbeing and behavior
Source: Glob Environ Change. 2022 May;74:102497. doi: 10.1016/j.gloenvcha.2022.102497 (PMC9664366; doi:10.1016/j.gloenvcha.2022.102497)
Supplement: Supplementary data 1 [file mmc1.docx]

**Appendix A**

Forest 404: Using a BBC drama series to explore the impact of nature’s changing soundscapes on human wellbeing and behavior

**This file includes:**

- Supplementary Text
- Figs. S1 to S2
- Tables S1 to S15

Supplementary Text

Online implementation

The full wording of the experimental instrument is available on the Open Science Framework (https://osf.io/p3gty). This open access document details the experimental procedure, pre-condition processes, and wording of all measures. It also includes information and consent sheets. Here we provide an overview of the participant experience.

Information and consent

The experiment was hosted on the Open University’s nQuire platform, an inquiry-based learning toolkit designed to support citizen-led science investigations. Calls to take part in the experiment were present at the end of every *Forest 404* podcast episode, which directed listeners to the *Forest 404* website. From there, participants were directed to the experiment page on nQuire. Respondents were initially presented with an information sheet detailing the experimental procedure, data handling, and process for withdrawal. They were asked to provide explicit online consent before being allowed to take part. Beyond the confirmation of consent, no questions within the experiment were mandatory.

Sound test

Upon beginning the experiment, participants were asked to complete a short sound test. This was designed to ensure their audio setup was optimized, they could hear the sounds they were about to play, and to familiarize them with the way the nQuire platform registers responses. Peak audio levels were normalized between conditions.

Vignette

Before listening to our nature-based experimental conditions, participants were asked to imagine a situation in which they felt stressed and cognitively fatigued. This approach was used due to the online nature of the experiment, which did not allow the measurement of actual stress inducement and recovery. A vignette-style mood induction was adapted in accordance with previous studies (Staats and Hartig, 2004) and participants were asked to imagine a stressful situation by reading the following paragraph:

“*Please imagine it’s been a difficult time for you lately. There’s been a lot going on and you’ve been feeling overstretched and on edge. You’ve also had trouble sleeping, found it difficult to concentrate, and feel irritable for no obvious reason. Now, to top it all off, you’ve just had an upsetting argument with a friend and feel very stressed out about it. You find yourself walking down a busy street and decide to sit on a bench and put your headphones on while you wait for a bus home.”*

To enhance immersion in this narrative, participants were asked to listen to a busy urban soundscape while reading the above passage. This soundscape lasted for 40 seconds and consisted of traffic and construction noise, with passing vehicles, jackhammers, and clanking scaffold poles also audible.

Stimuli and exposure

Following the mood induction, participants were asked to listen to one of our 40-second nature-based stimuli, randomly chosen from the pool of 36. They were instructed to listen to the sound in full first, with their eyes closed if possible. When the sound had finished playing, they were then asked to scroll down and answer the 8 questions which followed (detailed in ‘Measures development’ below). They were told they could play the sound again whilst answering the questions if they found it helpful. Participants then repeated this process for two more sounds, again chosen at random. To maximize relevance to the stressful vignette and to avoid possible ordering effects, we only considered data from respondents’ first sound in our analyses, creating a between-participant design.

Demographics and debrief

The experiment concluded with a series of demographic items. Upon completion of these sections, participants viewed a debrief screen which explained what would happen next in terms of analysis and publishing, and provided links to further information about the study.

Demographic questions

As highlighted in the main text, we included three demographic factors deemed to be important covariates in nature-health relationships in these analyses: sex, gender, and connection to nature. In addition, and in line with the UK’s Monitoring Engagement with the Natural Environment (MENE) survey, we also collected data on a further series of classification questions. Relationships with these factors and our questions of interest were inconsistent and they will be explored in further analyses beyond the scope of the current paper. The full list of demographic and behavior-based questions were:

1. What is your age?
2. Do you consider yourself to be: Female; Male; Another sex or gender?
3. Which of the following regions do you live in?
4. How would you characterize the surroundings in which you currently live?
5. How would you characterize the surroundings in which you grew up as a child?
6. Thinking about your place in the world, to what extent do you feel 'part of nature'?
7. How often do you watch or listen to nature programs on the TV or radio?
8. How often do you spend time in nature-based environments?
9. Are you a member of any nature-based organizations, such as the National Trust or RSPB for example?

Figure S1.


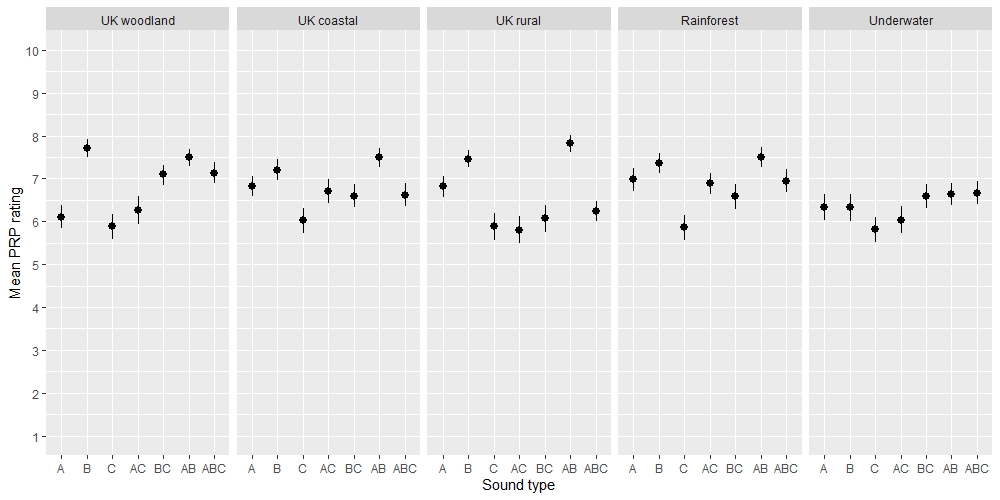


**Responses within biomes**. Mean perceived restorative potential ratings for individual sounds, displayed according to biome of origin. Confidence intervals (95%) are also displayed. Patterns for sound types were highly similar across biomes and were thus collapsed together for analyses presented in the main text. Inter-biome comparisons are beyond the scope of the current paper but will be explored in a subsequent publication.

Figure S2.


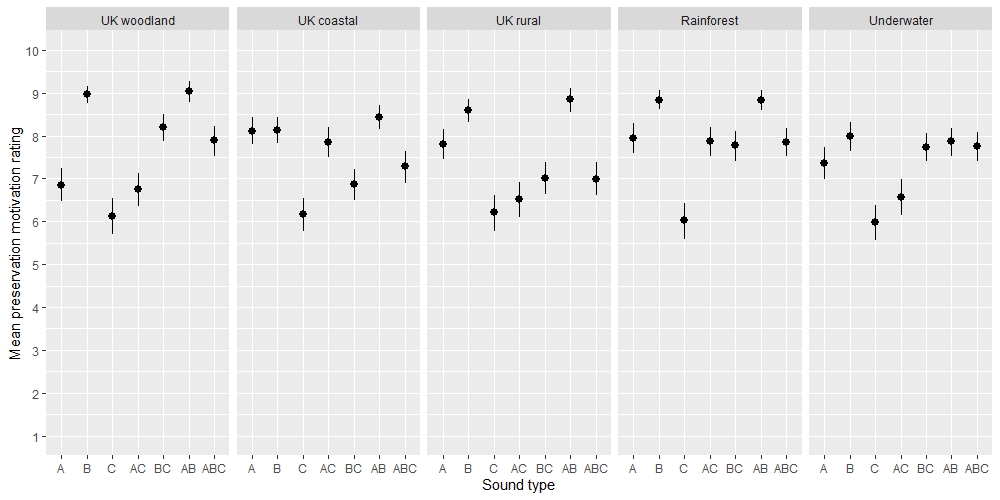


**Responses within biomes**. Mean preservation motivation ratings for individual sounds, displayed according to biome of origin. Confidence intervals (95%) are also displayed. Patterns for sound types were highly similar across biomes and were thus collapsed together for analyses presented in the main text. Inter-biome comparisons are beyond the scope of the current paper but will be explored in a subsequent publication.

Table S1.

| Variable | N | Male, N = 2618^1^ | Female, N = 4798^1^ | Another sex or gender, N = 53^1^ | |
| --- | --- | --- | --- | --- | --- |
| **Age** | 7347 |  |  |  | |
| 18-25 |  | 221 (8.6%) | 599 (13%) | 22 (42%) | |
| 26-35 |  | 460 (18%) | 936 (20%) | 13 (25%) | |
| 36-45 |  | 420 (16%) | 798 (17%) | 10 (19%) | |
| 46-55 |  | 654 (25%) | 1084 (23%) | 4 (7.7%) | |
| 56-65 |  | 528 (21%) | 931 (20%) | 3 (5.8%) | |
| 66-75 |  | 240 (9.3%) | 319 (6.8%) | 0 (0%) | |
| 76+ |  | 34 (1.3%) | 37 (0.8%) | 0 (0%) | |
| Prefer not to answer |  | 13 (0.5%) | 21 (0.4%) | 0 (0%) | |
| **Connection to nature** | 7425 | 7 (6, 8) | 7 (6, 9) | 7 (5, 8) | |
| ^1^Statistics presented: n (%); median (IQR) | | | | |  |

**Participant characteristics**. Note the relatively low prevalence of those reporting their sex as ‘Another sex or gender’. Total sample size = 7,596. Varying Ns are due to missing data for specific questions (no questions within the experiment were mandatory).

Table S2.

| **Characteristic** | **Beta** | **95% CI^1^** | **p-value** | **Fit** |
| --- | --- | --- | --- | --- |
| **(Intercept)** | 3.12 | 2.79, 3.44 | <0.001 |  |
| **Sound type** |  |  |  |  |
| Silence | – | – |  |  |
| A | 2.63 | 2.33, 2.93 | <0.001 |  |
| B | 3.26 | 2.95, 3.56 | <0.001 |  |
| C | 1.92 | 1.62, 2.23 | <0.001 |  |
| AC | 2.36 | 2.06, 2.67 | <0.001 |  |
| BC | 2.59 | 2.29, 2.89 | <0.001 |  |
| AB | 3.41 | 3.11, 3.72 | <0.001 |  |
| ABC | 2.71 | 2.40, 3.01 | <0.001 |  |
| **Connection to nature** | 0.13 | 0.11, 0.16 | <0.001 |  |
| **Age group** |  |  |  |  |
| 18-35 | – | – |  |  |
| 36+ | -0.01 | -0.11, 0.09 | 0.847 |  |
| **Sex** |  |  |  |  |
| Male | – | – |  |  |
| Female | -0.06 | -0.15, 0.04 | 0.250 |  |
| Another sex or gender | -0.29 | -0.85, 0.26 | 0.299 |  |
|  |  |  |  | R^2^ = 0.110**  95% CI [0.10, 0.12] |
| ^1^CI = Confidence Interval | | | |  |

**Hierarchies between soundscapes.** Multiple linear regression with perceived restorative potential as dependent variable. Silent sound type as reference condition. Beta represents unstandardized regression coefficients.

Table S3.

| **Characteristic** | **Beta** | **95% CI^1^** | **p-value** | **Fit** |
| --- | --- | --- | --- | --- |
| **(Intercept)** | 6.53 | 6.32, 6.74 | <0.001 |  |
| **Sound type** |  |  |  |  |
| AB | – | – |  |  |
| A | -0.78 | -0.95, -0.61 | <0.001 |  |
| B | -0.16 | -0.33, 0.02 | 0.074 |  |
| C | -1.49 | -1.66, -1.32 | <0.001 |  |
| AC | -1.05 | -1.22, -0.88 | <0.001 |  |
| BC | -0.82 | -1.00, -0.65 | <0.001 |  |
| ABC | -0.71 | -0.88, -0.53 | <0.001 |  |
| Silence | -3.41 | -3.72, -3.11 | <0.001 |  |
| **Connection to nature** | 0.13 | 0.11, 0.16 | <0.001 |  |
| **Age group** |  |  |  |  |
| 18-35 | – | – |  |  |
| 36+ | -0.01 | -0.11, 0.09 | 0.847 |  |
| **Sex** |  |  |  |  |
| Male | – | – |  |  |
| Female | -0.06 | -0.15, 0.04 | 0.250 |  |
| Another sex or gender | -0.29 | -0.85, 0.26 | 0.299 |  |
|  |  |  |  | R^2^ = 0.110**  95% CI [0.10, 0.12] |
| ^1^CI = Confidence Interval | | | |  |

**Comparisons**. Multiple linear regression with perceived restorative potential as dependent variable. AB sound type (abiotic and biotic combined) set as reference condition for sound type comparisons presented in the main text. Beta represents unstandardized regression coefficients.

**Table S4.**

| **Characteristic** | **Beta** | **95% CI^1^** | **p-value** | **Fit** |
| --- | --- | --- | --- | --- |
| **(Intercept)** | 5.04 | 4.83, 5.25 | <0.001 |  |
| **Sound type** |  |  |  |  |
| C | – | – |  |  |
| A | 0.71 | 0.53, 0.88 | <0.001 |  |
| B | 1.33 | 1.16, 1.50 | <0.001 |  |
| AB | 1.49 | 1.32, 1.66 | <0.001 |  |
| AC | 0.44 | 0.26, 0.61 | <0.001 |  |
| BC | 0.67 | 0.49, 0.84 | <0.001 |  |
| ABC | 0.78 | 0.61, 0.96 | <0.001 |  |
| Silence | -1.92 | -2.23, -1.62 | <0.001 |  |
| **Connection to nature** | 0.13 | 0.11, 0.16 | <0.001 |  |
| **Age group** |  |  |  |  |
| 18-35 | – | – |  |  |
| 36+ | -0.01 | -0.11, 0.09 | 0.847 |  |
| **Sex** |  |  |  |  |
| Male | – | – |  |  |
| Female | -0.06 | -0.15, 0.04 | 0.250 |  |
| Another sex or gender | -0.29 | -0.85, 0.26 | 0.299 |  |
|  |  |  |  | R^2^ = 0.110**  95% CI [0.10, 0.12] |
| ^1^CI = Confidence Interval | | | |  |

**Comparisons**. Multiple linear regression with perceived restorative potential as dependent variable. C sound type (poem only) set as reference condition for sound type comparisons presented in the main text. Beta represents unstandardized regression coefficients.

**Table S5.**

| **Characteristic** | **Beta** | **95% CI^1^** | **p-value** | **Fit** |
| --- | --- | --- | --- | --- |
| **(Intercept)** | 2.60 | 2.17, 3.03 | <0.001 |  |
| **Sound type** |  |  |  |  |
| Silence | – | – |  |  |
| A | 3.82 | 3.42, 4.21 | <0.001 |  |
| B | 4.70 | 4.31, 5.10 | <0.001 |  |
| C | 2.33 | 1.93, 2.72 | <0.001 |  |
| AC | 3.30 | 2.90, 3.70 | <0.001 |  |
| BC | 3.70 | 3.31, 4.10 | <0.001 |  |
| AB | 4.81 | 4.41, 5.20 | <0.001 |  |
| ABC | 3.70 | 3.30, 4.09 | <0.001 |  |
| **Connection to nature** | 0.13 | 0.10, 0.16 | <0.001 |  |
| **Age group** |  |  |  |  |
| 18-35 | – | – |  |  |
| 36+ | 0.24 | 0.11, 0.38 | <0.001 |  |
| **Sex** |  |  |  |  |
| Male | – | – |  |  |
| Female | 0.19 | 0.07, 0.32 | 0.003 |  |
| Another sex or gender | 0.08 | -0.63, 0.80 | 0.819 |  |
|  |  |  |  | R^2^ = 0.139**  95% CI [0.12, 0.15] |
| ^1^CI = Confidence Interval | | | |  |

**Preservation motivation**. Multiple linear regression with preservation motivation as the dependent variable. Silent sound type as reference condition. Beta represents unstandardized regression coefficients.

**Table S6.**

| **Characteristic** | **Beta** | **95% CI^1^** | **p-value** | **Fit** |
| --- | --- | --- | --- | --- |
| **(Intercept)** | 7.41 | 7.13, 7.68 | <0.001 |  |
| **Sound type** |  |  |  |  |
| AB | – | – |  |  |
| A | -0.99 | -1.22, -0.77 | <0.001 |  |
| B | -0.10 | -0.33, 0.12 | 0.4 |  |
| C | -2.48 | -2.71, -2.26 | <0.001 |  |
| AC | -1.51 | -1.74, -1.28 | <0.001 |  |
| BC | -1.10 | -1.33, -0.88 | <0.001 |  |
| ABC | -1.11 | -1.34, -0.88 | <0.001 |  |
| Silence | -4.81 | -5.20, -4.41 | <0.001 |  |
| **Connection to nature** | 0.13 | 0.10, 0.16 | <0.001 |  |
| **Age group** |  |  |  |  |
| 18-35 | – | – |  |  |
| 36+ | 0.24 | 0.11, 0.38 | <0.001 |  |
| **Sex** |  |  |  |  |
| Male | – | – |  |  |
| Female | 0.19 | 0.07, 0.32 | 0.003 |  |
| Another sex or gender | 0.08 | -0.63, 0.80 | 0.819 |  |
|  |  |  |  | R^2^ = 0.139**  95% CI [0.12, 0.15] |
| ^1^CI = Confidence Interval | | | |  |

**Comparisons**. Multiple linear regression with preservation motivation as dependent variable. AB sound type (abiotic and biotic combined) set as reference condition for sound type comparisons presented in the main text. Beta represents unstandardized regression coefficients.

**Table S7.**

| **Characteristic** | **Beta** | **95% CI^1^** | **p-value** | **Fit** |
| --- | --- | --- | --- | --- |
| **(Intercept)** | 4.92 | 4.65, 5.20 | <0.001 |  |
| **Sound type** |  |  |  |  |
| C | – | – |  |  |
| A | 1.49 | 1.26, 1.71 | <0.001 |  |
| B | 2.38 | 2.15, 2.60 | <0.001 |  |
| AB | 2.48 | 2.26, 2.71 | <0.001 |  |
| AC | 0.97 | 0.74, 1.20 | <0.001 |  |
| BC | 1.38 | 1.15, 1.60 | <0.001 |  |
| ABC | 1.37 | 1.14, 1.60 | <0.001 |  |
| Silence | -2.33 | -2.72, -1.93 | <0.001 |  |
| **Connection to nature** | 0.13 | 0.10, 0.16 | <0.001 |  |
| **Age group** |  |  |  |  |
| 18-35 | – | – |  |  |
| 36+ | 0.24 | 0.11, 0.38 | <0.001 |  |
| **Sex** |  |  |  |  |
| Male | – | – |  |  |
| Female | 0.19 | 0.07, 0.32 | 0.003 |  |
| Another sex or gender | 0.08 | -0.63, 0.80 | 0.819 |  |
|  |  |  |  | R^2^ = 0.139**  95% CI [0.12, 0.15] |
| ^1^CI = Confidence Interval | | | |  |

**Comparisons**. Multiple linear regression with preservation motivation as dependent variable. C sound type (poem only) set as the reference condition for sound type comparisons presented in the main text. Beta represents unstandardized regression coefficients.

**Table S8.**

| **Characteristic** | **Beta** | **95% CI^1^** | **p-value** | **Fit** |
| --- | --- | --- | --- | --- |
| **(Intercept)** | 4.78 | 4.52, 5.03 | <0.001 |  |
| **Memories** |  |  |  |  |
| None | – | – |  |  |
| Negative | -1.36 | -1.79, -0.92 | <0.001 |  |
| Mixed | 0.25 | 0.02, 0.48 | 0.037 |  |
| Positive | 1.94 | 1.78, 2.09 | <0.001 |  |
| **Connection to nature** | 0.08 | 0.05, 0.11 | <0.001 |  |
| **Age group** |  |  |  |  |
| 18-35 | – | – |  |  |
| 36+ | 0.10 | -0.04, 0.24 | 0.172 |  |
| **Sex** |  |  |  |  |
| Male | – | – |  |  |
| Female | 0.07 | -0.06, 0.21 | 0.284 |  |
| Another sex or gender | 0.05 | -0.64, 0.74 | 0.890 |  |
|  |  |  |  | R^2^ = 0.240**  95% CI [0.21, 0.26] |
| ^1^CI = Confidence Interval | | | |  |

**The moderating role of memories**. Multiple linear regression with perceived restorative potential as dependent variable. ‘No memories’ category as reference condition. Beta represents unstandardized regression coefficients. Note that the format of our memory-based question prevented us from interpreting memories for our combined soundscapes (we could not determine which component the memory related to), so for these analyses we focused on single component soundscapes (A, B, or C) collapsed together.

**Table S9.**

| **Characteristic** | **Beta** | **95% CI^1^** | **p-value** | **Fit** |
| --- | --- | --- | --- | --- |
| **(Intercept)** | 4.98 | 4.62, 5.34 | <0.001 |  |
| **Memories** |  |  |  |  |
| None | – | – |  |  |
| Negative | -0.90 | -1.51, -0.29 | 0.004 |  |
| Mixed | 0.34 | 0.01, 0.67 | 0.043 |  |
| Positive | 2.33 | 2.12, 2.55 | <0.001 |  |
| **Connection to nature** | 0.08 | 0.04, 0.13 | <0.001 |  |
| **Age group** |  |  |  |  |
| 18-35 | – | – |  |  |
| 36+ | 0.33 | 0.13, 0.52 | 0.001 |  |
| **Sex** |  |  |  |  |
| Male | – | – |  |  |
| Female | 0.37 | 0.18, 0.56 | <0.001 |  |
| Another sex or gender | 0.74 | -0.21, 1.70 | 0.127 |  |
|  |  |  |  | R^2^ = 0.182**  95% CI [0.16, 0.20] |
| ^1^CI = Confidence Interval | | | |  |

**Memories and preservation motivation**. Multiple linear regression with preservation motivation as dependent variable. ‘No memories’ category as reference condition. Beta represents unstandardized regression coefficients.

**Table S10.**

| **Characteristic** | **Beta** | **95% CI^1^** | **p-value** | **Fit** |
| --- | --- | --- | --- | --- |
| **(Intercept)** | 5.08 | 4.71, 5.44 | <0.001 |  |
| **Memories** |  |  |  |  |
| No | – | – |  |  |
| Yes | 1.11 | 0.80, 1.41 | <0.001 |  |
| **Sound type** |  |  |  |  |
| A | – | – |  |  |
| B | 0.51 | 0.13, 0.89 | 0.009 |  |
| C | -0.78 | -1.11, -0.45 | <0.001 |  |
| **Age group** |  |  |  |  |
| 18-35 | – | – |  |  |
| 36+ | 0.05 | -0.09, 0.20 | 0.471 |  |
| **Sex** |  |  |  |  |
| Male | – | – |  |  |
| Female | 0.00 | -0.14, 0.14 | 0.985 |  |
| Another sex or gender | -0.32 | -1.04, 0.40 | 0.386 |  |
| **Connection to nature** | 0.09 | 0.05, 0.12 | <0.001 |  |
| **Memory * sound type interactions** |  |  |  |  |
| Memories = Yes * sound type B | 0.17 | -0.25, 0.59 | 0.437 |  |
| Memories = Yes * sound type C | 0.58 | 0.19, 0.96 | 0.003 |  |
|  |  |  |  | R^2^ = 0.171**  95% CI [0.15, 0.19] |
| ^1^CI = Confidence Interval | | | |  |

**Memory x sound type interactions for restorative potential**. Multiple linear regression with perceived restorative potential as dependent variable. ‘No memories’ and sound type ‘A’ (abiotic sounds) as reference categories and memory x sound type interactions included. Beta represents unstandardized regression coefficients.

**Table S11.**

| **Memory group** | | **Sound type** | **Fitted value** | **SE** | **CI lower** | **CI upper** |  |
| --- | --- | --- | --- | --- | --- | --- | --- |
| No | A | | 5.71 | 0.14 | 5.44 | 5.99 |  |
| Yes | A | | 6.82 | 0.07 | 6.69 | 6.95 |  |
| No | B | | 6.22 | 0.13 | 5.96 | 6.49 |  |
| Yes | B | | 7.50 | 0.07 | 7.37 | 7.63 |  |
| No | C | | 4.94 | 0.09 | 4.76 | 5.12 |  |
| Yes | C | | 6.62 | 0.08 | 6.47 | 6.77 |  |

**Fitted values.** Estimated marginal means from the model described in Table S10 (perceived restorative potential predicted by memory group and sound type) and shown in Figure 5A in the main text.

**Table S12.**

| Characteristic | Beta | 95% CI^1^ | p-value | Fit |
| --- | --- | --- | --- | --- |
| **(Intercept)** | 5.58 | 5.09, 6.06 | <0.001 |  |
| **Memories** |  |  |  |  |
| No | – | – |  |  |
| Yes | 1.21 | 0.80, 1.61 | <0.001 |  |
| **Sound type** |  |  |  |  |
| A | – | – |  |  |
| B | 0.91 | 0.41, 1.42 | <0.001 |  |
| C | -1.65 | -2.09, -1.21 | <0.001 |  |
| **Age group** |  |  |  |  |
| 18-35 | – | – |  |  |
| 36+ | 0.29 | 0.10, 0.49 | 0.003 |  |
| **Sex** |  |  |  |  |
| Male | – | – |  |  |
| Female | 0.27 | 0.09, 0.46 | 0.004 |  |
| Another sex or gender | 0.35 | -0.60, 1.29 | 0.470 |  |
| **Connection to nature** | 0.10 | 0.05, 0.14 | <0.001 |  |
| **Memory * sound type interactions** |  |  |  |  |
| Memories = Yes * sound type B | 0.00 | -0.56, 0.56 | 0.994 |  |
| Memories = Yes * sound type C | 0.74 | 0.23, 1.26 | 0.005 |  |
|  |  |  |  | R^2^ = 0.199**  95% CI [0.17, 0.22] |
| ^1^CI = Confidence Interval | | | |  |

**Memory x sound type interactions for preservation motivation**. Multiple linear regression with preservation motivation as dependent variable. ‘No memories’ and sound type ‘A’ (abiotic sounds) as reference categories and memory x sound type interactions included. Beta represents unstandardized regression coefficients.

**Table S13.**

| **Memory group** | **Sound type** | **Fitted value** | **SE** | **CI lower** | **CI upper** |  |
| --- | --- | --- | --- | --- | --- | --- |
| No | A | 6.64 | 0.19 | 6.27 | 7.00 |  |
| Yes | A | 7.84 | 0.09 | 7.67 | 8.01 |  |
| No | B | 7.55 | 0.18 | 7.20 | 7.89 |  |
| Yes | B | 8.76 | 0.09 | 8.58 | 8.93 |  |
| No | C | 4.99 | 0.12 | 4.74 | 5.23 |  |
| Yes | C | 6.94 | 0.10 | 6.73 | 7.14 |  |

**Fitted values**. Estimated marginal means from the model described in Table S12 (preservation motivation predicted by memory group and sound type) shown in Figure 5B in the main text.

**Table S14.**

| Variable | *M* | | *SD* | 1 | | 2 | | 3 | | 4 | |  |
| --- | --- | --- | --- | --- | --- | --- | --- | --- | --- | --- | --- | --- |
|  |  |  | | |  | |  | |  | |  | |
| 1. PRP (combined) | 6.61 | 2.09 | | |  | |  | |  | |  | |
|  |  |  | | |  | |  | |  | |  | |
| 2. PRP (single item) | 6.47 | 2.17 | | | .88** | |  | |  | |  | |
|  |  |  | | | [.87, .88] | |  | |  | |  | |
|  |  |  | | |  | |  | |  | |  | |
| 3. Fascination | 6.38 | 2.43 | | | .89** | | .64** | |  | |  | |
|  |  |  | | | [.88, .89] | | [.63, .66] | |  | |  | |
|  |  |  | | |  | |  | |  | |  | |
| 4. Being away | 6.98 | 2.39 | | | .92** | | .75** | | .73** | |  | |
|  |  |  | | | [.92, .93] | | [.74, .76] | | [.72, .74] | |  | |
|  |  |  | | |  | |  | |  | |  | |
| 5. Preservation motivation | 7.48 | 2.78 | | | .64** | | .55** | | .57** | | .60** | |
|  |  |  | | | [.63, .65] | | [.53, .56] | | [.56, .59] | | [.58, .61] | |
|  |  |  | | |  | |  | |  | |  | |

*Note.* *M* and *SD* are used to represent mean and standard deviation, respectively. Values in square brackets indicate the 95% confidence interval for each correlation. The confidence interval is a plausible range of population correlations that could have caused the sample correlation. ** indicates *p* < .01.

**Correlations among our dependent variables**. ‘*PRP (combined)*’ is our composite measure of perceived restorative potential described in the main text, created by collapsing our ‘*PRP (single)*’, ‘*Fascination*’, and ‘*Being away*’ items.

**Table S15.**

| **Outcome** | **Predictor** | **Effects pathway** | **Estimate** | **95% CIs^1^** |
| --- | --- | --- | --- | --- |
|  |  |  |  |  |
| **Perceived restorative potential (PRP)** | **Sound type A vs C** | Direct (Total) | 0.34*** | 0.20, 0.49 |
|  | **Sound type B vs C** | Direct (Total) | 1.01*** | 0.86, 1.17 |
|  | **Memories Yes vs. No** | Direct (Total) | 1.45*** | 1.27, 1.62 |
|  |  | Total variance explained^2^ (R^2^) | 21% |  |
|  |  |  |  |  |
| **Preservation motivation** | **Sound type A vs C** | Direct | 0.88*** | 0.71, 1.07 |
|  |  | Indirect via PRP | 0.25*** | 0.14, 0.35 |
|  |  | Total effect | 1.13*** | 0.92, 1.35 |
|  |  | **Proportion mediated** | **0.22** |  |
|  | **Sound type B vs C** | Direct | 1.36*** | 1.19, 1.54 |
|  |  | Indirect via PRP | 0.72*** | 0.61, 0.86 |
|  |  | Total effect | 2.08*** | 1.89, 2.28 |
|  |  | **Proportion mediated** | **0.35** |  |
|  | **Memories ‘yes’ vs ‘no’** | Direct | 0.53*** | 0.33, 0.74 |
|  |  | Indirect via PRP | 1.04*** | 0.90, 1.18 |
|  |  | Total effect | 1.56*** | 1.33, 1.80 |
|  |  | **Proportion mediated** | **0.67** |  |
|  | **Restorative potential** | Direct (total) | 0.72*** | 0.67, 0.76 |
|  |  | Total variance explained^2^ (R^2^) | 48% |  |
|  |  |  |  |  |
| **Covariances** | **Memories ↔ sound type A** | Direct | 0.053*** | 0.045, 0.061 |
|  | **Memories ↔ sound type B** | Direct | 0.049*** | 0.041, 0.056 |
|  |  |  |  |  |
| ^1^CI = Confidence Interval.  ^2^Includes age, sex, and connection to nature as covariates.  Coefficients are bootstrapped (1,000 samples).  *p*-values denoted by asterisks *** *p*<0.001. | | | | |

**Mediation model**. Tabular results of mediation analysis shown in Figure 6 in the main text and testing perceived restorative potential as mediator of the effects of memories and sound type on preservation motivation.
